# Differences between men and women in self-reported body mass index and its relation to drug use

**DOI:** 10.1186/1747-597X-9-1

**Published:** 2014-01-02

**Authors:** Pablo Vera-Villarroel, José A Piqueras, Walter Kuhne, Pim Cuijpers, Annemieke van Straten

**Affiliations:** 1 School of Psychology, Universidad de Santiago de Chile (USACH), Avenida Ecuador 3650, tercer Piso, Estación Central, Santiago de Chile, Chile; 2 Department of Health Psychology, Universidad Miguel Hernández de Elche, Edif, Altamira, Avda. de la Universidad s/n, 03202, Elche, Alicante, Spain; 3 Department of Clinical Psychology, VU University Amsterdam, Van der Boechorststraat 1, 1081 BT Amsterdam, The Nederlands

**Keywords:** Overweight, Obesity, Drugs, Health-risk behaviors, University students

## Abstract

**Background:**

Obesity is a public health problem of alarming proportions, including among the university population in Latin America. The purpose of this study was to determine the relation between the self-reported body mass index and the associated drug use and health-risk behaviors.

**Methods:**

We performed a cross-sectional, descriptive study of 3,311 Chilean university students (17–24 years). The variables weight, height, frequency of physical activity, diet quality index, and drug use were evaluated by way of a self-report questionnaire.

**Results:**

16.7% of students were overweight and 2.1% were obese. Higher rates of overweight and obesity were observed in the men compared to women. There was a significant but moderate association between self-perceived obesity and being men and higher age, and just low with greater use of analgesics and tranquilizers with or without a prescription.

**Conclusions:**

The punctual prevalence rates of self-reported obesity, in this sample, are consistent with other Latin American studies. The risk behaviors associated with perceived obesity in terms of gender, particularly the different pattern of drug use, highlight the importance of considering gender when designing strategies to promote health in a university setting.

## Background

The increase and high prevalence of obesity has led the World Health Organization to consider it a chronic disease of epidemic proportions [1]. This condition is a significant modifiable risk factor for most of the main causes of disability and mortality, such as cardiovascular diseases, diabetes mellitus and osteoarthritis, in both developed [2-4] and developing countries [5]. It has been related to increased symptoms of anxiety and depression in adolescents and college students [6-8], and to greater comorbidity with affective, anxiety, and substance disorders, as well as some personality disorders [9,10].

Although there are studies in adult and pediatric populations, and international university populations [11-16], much less evidence exists regarding Latin America [17]. In Chile, the National Health Survey 2003 (ENS) provided prevalence data for those between the17 and 24 years of age, which indicated that males were 14% overweight, 10% obese and 0.3% morbidly obese, and females were 19.6%, 7.1%, and 1.1%, respectively.

Despite the great number of recent studies on obesity and associated conditions in the Latin American population [5,17-28], fewer studies explore its relation with other health-risk behaviors, including smoking, alcohol and other drug use [1,5].

Specifically, university population studies show a higher prevalence of legal and illegal drug use than in other populations [29], with implications for public health problems, as well as the relationship between drug use and academic performance. Recent studies have shown a relationship between drug use and sexual in dating violence in college and university students. This justifies the need for a more specific analysis between various health problems in young people who may be linked to drug use and associated factors [30].

Although very few studies have studied the connection between drug use and obesity or other associated indicators (for a recent review about the link between obesity, weight, and drug abuse see Nolan [31], those that have done so have reported a positive relation between alcohol and tobacco use, poor diet, and body dissatisfaction, and overweight/obesity [23,32]. There are even fewer studies dealing with the issue of possible gender differences. It is well known that there are differences between men and women in the prevalence of mental disorders [23-35], physical diseases [28,36], and risk factors for sexually transmitted diseases, including HIV [37-39]. However, there is much less information with respect to gender differences and weight-related risk factors, and even less on associated factors such as lifestyle or drug use.

Recently, there is some evidence showing different effects of drug use, and BMI in men and women, even different effects depending on the type of drug. Some of these studies have examined the relation between the Body Mass Index (BMI) and substance-related disorders [10,40], indicating contradictory findings regarding the linking between overweight/obesity and a greater risk of drugs abuse and dependence throughout life in men or women. However, the specific available literature on these topics shows inconclusive evidence. Epidemiological studies seem to suggest that, especially in women, a moderate alcohol consumption can protect against obesity, whereas that a growing consumption of alcohol would increase the risk of obesity [41]. In relation to consumption of cannabis and BMI, literature is contradictory showing even that there is no relationship between this consumption and weight in women [42]. A two years follow-up study showed no association between drug use in adolescence and subsequent body weight [43]. Moreover, in men, who took drugs, it was found an association with a lower BMI, but not in women [44]. It has also been reported that overweight men have higher rates of alcohol dependence, whereas the opposite result is found in overweight women, with a lower incidence of alcohol dependence [40]. As can be seen, previous studies show differences in the associations between drug use, and BMI and also that there are gender differences. Additionally, following Nolan [31], most of the reported studies are based on diagnostic categories, and often do not consider the use of drugs and foods.

Given the relevance of the period for young people at university, where poor nutrition, poor psychical exercise habits and access to drugs may increase, studies targeting this population are needed to analyze the possible related variables both in lifestyle and gender differences and to suggest new health policies. However, more evidence is needed from different cultures that permit have specific evidence about the relationship between BMI and drug.

As a result, on the basis of the accumulated evidence reviewed above, the purpose of the present study was to examine the relation between the self-reported BMI and the various risk-associated behaviors, including lack of physical exercise, low quality diet, and especially legal and illegal drug use in both men and women. Specifically, we analyzed the relationship between BMI and drug consumption.

## Methods

### Participants

The data analyzed for this study were taken from the Quality of Life and Health Behaviors Survey, a cross-sectional descriptive study by means of a survey that included a randomly selected sample representative of the population of students at the University de Santiago de Chile. Respondents were enrolled on a variety of programs, including Administration and Economy, Science, Medical Sciences, Humanities, Engineering, Technology, Chemistry and Biology, Architecture and Bachelor program. They came from 166 of the 346 municipalities (comunas) in which Chile is divided, so that the socio-economic status of the sample can be considered broad and representative of universities from this country. The entire procedure and instruments were reviewed and approved by the Ethics Committee of the University of Santiago de Chile.

### Study sample

The sample comprised 3,311 university students, 1,818 (54.9%) men and 1,493 (45.1%) women, whose mean age was 19.91 years (SD = 1.74; range = 17–24). The questionnaires were administered during classroom visits. Consent in writing was requested. Therefore, each participant signed an informed written consent that indicated the objectives and responsible researchers of the study. Participation, therefore, was voluntary, anonymous and confidential. Interviewers were psychologists and they were trained in the administration of the survey by the researchers prior to the administration of the survey 442 questionnaires were incomplete or incorrectly answered and were eliminated.

### Measures

The Quality of Life and Health Behaviors Survey includes general information, such as age, gender, study program, body weight and height, and a range of measures of perceived stress and health risk behaviors, such as frequency of drug use, physical activity, and eating vegetables/fruits. This self-report instrument was developed ad hoc for this investigation, made up of questions extracted from the questionnaire developed by the National Council on Narcotics Prevention (CONACE) for the National Study of Drugs Use in the General Chilean Population 2008 [45]:

- Consumption of legal and illegal drugs. The items were selected from the same questionnaire developed by the CONACE. It attempts to measure consumption of legal and illegal drugs by means of the following question: When was the last time you tried any of these drugs? Responders can rated on a scale from 1 to 4 (“1 = Never”, “2 = More than 1 year ago”, “3 = More than a month, but less than one year” and “4 = During the past 30 days”. These questions are grouped into three factors: high-frequency use drugs (alcohol, tobacco and marijuana), medium-frequency use drugs (tranquilizers with or without a prescription and over-the-counter analgesics), and low-frequency use drugs (illegal drugs –cocaine, heroin, ecstasy, etc., except marijuana).

- Nutrition aspects and physical activity were assessed using five options from “Never = 1” to “Daily = 5” to evaluate the frequency of breakfast, lunch, eating fruit and vegetables, and doing exercise. The Diet Quality Index consisted of the average score of the first three items. Physical activity was based on the fourth item.

- The weight categories were calculated from self-reported height and weight and based on body mass index. We followed criteria of the World Health Organization (World Health Organization, WHO) [46] for BMI categories: underweight (BMI ≤ 18.49 kg m-2), normal weight (BMI = 18.50 to 24.90 kg m-2), overweight (BMI = 25.00 to 29.90 kg m-2) and obesity (BMI ≥ 30.00 kg m-2).

Final questionnaire had 120 items. For a more detailed description of this instruments see the Measures section in Piqueras et al. [21].

### Data analysis

The variables were analyzed using descriptive statistics and presented as means (M) and standard deviation (SD) in the case of the continuous variables, and as percentages in the case of the nominal variables. The differences between groups were established by means of the Student’s t-test, ANOVAs for continuous variables and χ2 for categorical variables. For the comparisons between the four categories of BMI (underweight, normal, overweight and obese), the data were first analyzed for each dependent variable (physical activity, diet quality index, and overall, high-, medium-, and low-frequency drug use) by a Factorial Analysis of Variance (ANOVA), taking weight categories, age and sex as fixed factors. Given that we found some interaction effects of sex with weight categories and of sex, age and weight categories for some tests, we conducted the ANOVAs separately for men and women. In addition to this, Scheffé’s tests were used to determine post-hoc pair-wise comparisons. In order to establish the association between the BMI and sex, age, the diet quality index, physical exercise and drug use were analyzed by multiple regression analysis for the total sample and for men and women separately. As a previous step to the different regression analysis we analyzed the correlations between all predictor or independent variables, in order to ensure that these bivariate correlations were less than 0.70 and thus to rule out multicollinearity [47]. Furthermore, we computed analysis of multicollinearity which included obtaining Variance Inflation Factor (VIF) and tolerance value (1-R^2^). For all variables VIF value was less than 10 and tolerances were all above 0.1. It was not considered then the exclusion of any variable for this reason. The results were considered statistically significant with a significance level of ≤ 0.01 due to our sample was large and we contrasted very many hypothesis tests. The software IBM SPSS 19.0 for Windows was used for the data analyses.

## Results

Table 1 shows the general characteristics of the participants with respect to the different variables considered, as well as the gender differences.

**Table 1:** Mean values and standard deviations (SD) of the anthropometric characteristics, diet quality, physical activity and drug use of university students

	**Range**	**Total (N = 3311)**	**Men (N = 1818)**	**Women (N = 1493)**	**t**_**3309**_	***p *** **Value**
**Age (years)**	17-24	19.91 (1.74)	19.82 (1.73)	20.03 (1.74)	-3.489	0.001***
**Weight (kg)**	35-170	65.32 (11.72)	71.65 (10.38)	57.61 (8.06)	42.749	0.001***
**Height (m)**	1,40-1,97	1.69 (0.91)	1.75 (0.06)	1.61 (0.06)	61.930	0.001***
**Body Mass Index (BMI)**	14,19-63,28	22.86 (3.00)	23.46 (3.00)	22.14 (2.84)	12.931	0.001***
**Physical activity (frequency)**	1-5	2.87 (1.11)	3.14 (1.06)	2.53 (1.08)	16.267	0.001***
**Diet Quality Index (frequency)**	4-15	12.20 (2.12)	12.20 (2.12)	12.20 (2.12)	-0.041	0.970
**Overall drug use**	1-4	1.60 (0.37)	1.61 (0.36)	1.59 (0.37)	1.568	0.120
**High-frequency drug use**	1-4	2.54 (0.88)	2.60 (0.88)	2.48 (0.88)	4.093	0.001^***^
**Medium-frequency drug use**	1-4	1.23 (0.47)	1.20 (0.43)	1.28 (0.52)	-4.823	0.001^***^
**Low-frequency drug use**	1-4	1.04 (0.17)	1.05 (0.18)	1.03 (0.15)	3.158	0.002^***^

*p < 0.05, **p < 0.01, ***p < 0.005.

With regard to weight categories based on BMI (Table 2), a prevalence of 16.7% overweight and 2.1% obesity was found. The analysis by gender revealed that men’s overweight and obesity rates were double those of women (24.5% and 11.6%, respectively). Among the female students, values of normal weight were more frequently found, but there was also underweight compared to the men (χ2 = 108.932, df = 3; p = 0.001).

**Table 2:** **Percentage distribution of the weight categories based on body mass index according to the WHO**[46]**of university students**

	**Underweight**	**Normal**	**Overweight**	**Obesity**
**Male**	1.8	73.7	21.9	2.6
**Female**	5.1	83.3	10.3	1.3
**Total**	3.3	78.0	16.7	2.1

The results for the total sample show significant differences among the weight categories (obesity, overweight, normal weight and underweight) based on the BMI for age, physical activity, and overall, high-, medium-, and low-frequency drug use (See Table 3). Post-hoc Scheffe’s tests confirmed that this was due to the obese group had more age and overall use of drugs than the underweight group; more age, use of medium-frequency and low-frequency drugs than the normal group; and more use of medium-frequency drugs than the overweight group. For the overweight group, post-hoc comparison showed this group had more age, physical activity, overall drugs use and high-frequency drug use than the underweight and normal groups. Regarding the normal group, it displayed more psychical activity and use of high-frequency drugs than the underweight group (see Table 3).

**Table 3:** Relation between weight categories and age, diet quality index, smoking habit, physical activity and current drug use based on BMI classification

	**Underweight U M (SD)**	**Normal N M (SD)**	**Overweight Ov M (SD)**	**Obesity Ob M (SD)**	**Total M (SD)**	**df**	**F**	***p***	***Post hoc***					
									**U v N**	**U v Ov**	**U v Ob**	**N v Ov**	**N v Ob**	**Ov v Ob**
**Age (years)**	19.72 (1.71)	19.83 (1.71)	20.26 (1.80)	20.54 (1.97)	19.91 (1.74)	3, 3299	12.547	0.001***		*	*	***	*	
**Physical activity (frequency)**	2.60 (1.14)	2.87 (1.13)	2.91 (1.05)	2.65 (0.94)	2.86 (1.11)	3, 3262	3.341	0.018*	*	**		*		
**Diet Quality Index (frequency)**	12.36 (2.22)	12.24 (2.11)	12.05 (2.14)	12.04 (1.89)	12.21 (2.12)	3, 3264	1.392	0.243						
**Overall drug use**	1.53 (0.40)	1.60 (0.36)	1.65 (0.36)	1.72 (0.51)	1.60 (0.37)	3, 3226	6.961	0.001***		*	*	*		
**High-frequency drug use**	2.27 (0.92)	2.53 (0.88)	2.65 (0.88)	2.63 (0.94)	2.54 (0.88)	3, 3260	6.797	0.001**^*^	*	**		*		
**Medium-frequency drug use**	1.27 (0.55)	1.22 (0.46)	1.24 (0.47)	1.45 (0.74)	1.23 (0.47)	3, 3267	5.293	0.001**					**	*
**Low-frequency drug use**	1.03 (0.12)	1.04 (0.16)	1.05 (0.17)	1.09 (0.35)	1.04 (0.17)	3, 3242	3.028	0.028*					*	

Total sample.

Note: Significance levels for each ANOVA and Scheffé’s post-hoc comparison are depicted by: *p < 0.05, **p < 0.01, ***p < 0.005.

In the sample of male participants, statistically significant differences were found among weight categories for age, physical activity, diet quality index and drug use, particularly medium- and low-frequency drug use. Additionally, the Scheffé’s post-hoc comparisons indicated that students in the overweight category were older than those who were at normal weight; that the students who were obese did less physical exercise than those who had normal weight, and had greater use of medium-frequency (such as analgesics and tranquilizers) and high-frequency drugs (illegal drugs, except marijuana) (see Table 4).

**Table 4:** Relation between weight categories and age, diet quality index, smoking habit, physical activity and current drug use based on BMI classification

	**Underweight U M (SD)**	**Normal N M (SD)**	**Overweight Ov M (SD)**	**Obesity Ob M (SD)**	**Total M (SD)**	**df**	**F**	***p***	***Post hoc***					
									**U v N**	**U v Ov**	**U v Ob**	**N v Ov**	**N v Ob**	**Ov v Ob**
**Age (years)**	19.30 (1.61)	19.71 (1.69)	20.15 (1.82)	20.29 (1.77)	19.82 (1.73)	3, 1812	8.727	0.001***				***		
**Physical activity (frequency)**	2.85 (1.09)	3.19 (1.07)	3.07 (1.02)	2.67 (0.95)	3.14 (1.06)	3, 1792	5.466	0.001**					**	
**Diet Quality Index (frequency)**	12.45 (2.03)	12.28 (2.11)	11.97 (2.15)	11.64 (1.96)	12.20 (2.12)	3, 1794	3.585	0.013*						
**Overall drug use**	1.54 (0.46)	1.60 (0.35)	1.65 (0.37)	1.73 (0.53)	1.61 (0.36)	3, 1779	4.193	0.006**						
**High-frequency drug use**	2.34 (1.09)	2.58 (0.86)	2.67 (0.89)	2.69 (0.94)	2.60 (0.88)	3, 1794	2.136	0.094						
**Medium-frequency drug use**	1.22 (0.62)	1.18 (0.40)	1.23 (0.48)	1.38 (0.70)	1.19 (0.43)	3, 1801	4.662	0.003**					**	
**Low-frequency drug use**	1.05 (0.18)	1.04 (0.16)	1.05 (0.18)	1.12 (0.40)	1.05 (0.18)	3, 1788	2.895	0.034*					*	

Men.

Note: Significance levels for each ANOVA and Scheffé’s post-hoc comparison are depicted by: *p < 0.05, **p < 0.01, ***p < 0.005.

Among the female participants, statistically significant differences were found for age and drug use, namely high- and medium-frequency drug use. Specifically, the Scheffé’s post-hoc comparisons found that there were significant differences related to a greater age of the students with obesity compared to the normal and underweight groups, and a greater age of the group with overweight compared to those with normal weight. Furthermore, the female students in the overweight category scored significantly higher in high-frequency drug use compared to those in the underweight category, whereas, for medium-frequency drug use, females in the obesity category showed higher scores than those with normal weight (see Table 5).

**Table 5:** Relation between weight categories and age, diet quality index, smoking habit, physical activity and current drug use based on BMI classification

	**Underweight U M (SD)**	**Normal N M (SD)**	**Overweight Ov M (SD)**	**Obesity Ob M (SD)**	**Total M (SD)**	**df**	**F**	***p***	***Post hoc***					
									**U v N**	**U v Ov**	**U v Ob**	**N v Ov**	**N v Ob**	**Ov v Ob**
**Age (years)**	19.91 (1.74)	19.96 (1.72)	20.53 (1.74)	21.15 (2.30)	20.03 (1.74)	3, 1483	7.892	0.001***			*	**	*	
**Physical activity (frequency)**	2.49 (1.15)	2.53 (1.09)	2.51 (1.04)	2.60 (0.94)	2.53 (1.08)	3, 1466	0.089	0.966						
**Diet Quality Index (frequency)**	12.31 (2.31)	12.18 (2.11)	12.29 (2.09)	13.00 (1.30)	12.21 (2.11)	3, 1466	1.135	0.334						
**Overall drug use**	1.52 (0.38)	1.59 (0.37)	1.64 (0.35)	1.71 (0.47)	1.59 (0.37)	3, 1443	2.210	0.085						
**High-frequency drug use**	2.24 (0.84)	2.47 (0.89)	2.62 (0.84)	2.48 (0.96)	2.47 (0.88)	3, 1462	3.055	0.028*		*				
**Medium-frequency drug use**	1.29 (0.52)	1.27 (0.51)	1.27 (0.46)	1.60 (0.82)	1.28 (0.52)	3, 1462	2.688	0.045*					*	
**Low-frequency drug use**	1.02 (0.08)	1.03 (0.16)	1.03 (0.11)	1.03 (0.11)	1.03 (0.15)	3, 1450	0.068	0.977						

Women.

Note: Significance levels for each ANOVA and Scheffé’s post-hoc comparison are depicted by: *p < 0.05, **p < 0.01, ***p < 0.005.

The results of the multiple regression analysis showed that sex, (d = -0.44), age (0.27), and use of medium-frequency drugs (d = 0.07) correlated positively with BMI. The multiple regression model used on the sample of male students indicated that age and medium-frequency drug use were positively and directly related to BMI (d = 0.23 and d = 0.07, respectively). As for the multiple regression model applied to the sample of female students, the only variable that showed significant correlation with BMI was age (d = 0.15) (See Table 6).

**Table 6:** Multiple regression analysis of the determining factors of the body mass index (BMI) of university students

**Dependent variable**	**Prognostic variable**		**Total**^**a**^			**Men**^**b**^			**Women**^**c**^	
		***β***	**T**_**3202**_	**p**	***β***	**t**	**p**	***β***	**T**	**p**
**Body Mass Index (BMI)**	Constant		25.87	0.001^*^		17.61	0.001^*^		15.80	0.001^***^
	Sex	-0.23	-12.63	0.001^*^						
	Age (years)	0.13	7.76	0.001^***^	0.15	6.43	0.001^***^	0.11	4.29	0.001^***^
	Physical activity (frequency)	-0.02	-0.98	0.32	-0.03	-1.09	0.274	-0.01	-0.07	0.940
	Diet Quality Index (frequency)	-0.02	-0.99	0.32	-0.04	-1.81	0.070	0.02	0.59	0.553
	High-frequency drug use	0.02	1.42	0.16	0.01	0.24	0.810	0.05	1.77	0.076
	Medium-frequency drug use	0.04	2.07	0.03^*^	0.05	2.02	0.043^*^	0.03	1.08	0.280
	Low-frequency drug use	0.01	0.83	0.41	0.02	0.95	0.341	-0.01	-0.08	0.932

^a^r = 0.26; r^2^ = 0.07. ^b^r = 0.18; r^2^ = 0.03. ^c^r = 0.13; r^2^ 0.02.

*p < 0.05, **p < 0.01, ***p < 0.005.

## Discussion and conclusions

The levels of BMI of the studied sample were noteworthy from a public health point of view. 16.7% of the students reported being overweight, and 2.1% of being obese. These values are lower than the rates of reported obesity prevalence for the general adult population in Latin America, which is between 9.9% and 35.7% [18,19,48,49] and in Spain, where a higher prevalence of obesity (14.5-22.3%) and overweight (18.7-37.6%) has been reported [20,49]. These prevalence rates were equivalent to the rates of overweight (16.5%), but lower than those of obesity (9.25%) as reported by the last ENS of the Government of Chile in 2003 [20]. These data are consistent with other studies that indicate that overweight and obesity are up to 2 times less frequent in people with a higher level of education [49]. These percentages are very similar to the results found by studies of Spanish-speaking university populations (overweight: 10.9-21.8% and obesity: 1.3-18%) [11,17,23,50] or the study by Lowry et al. of American university populations (obesity and overweight: 27.4%) [12].

Our study also showed that the mean BMI was significantly higher in the male students than in the females in the general sample and that there was a higher obesity and overweight rate among the males (24.5% compared to 11.6% of the women). Our data indicates that the problem is more significant for male students than for females, unlike the ENS Chile 2003 [20], which provided similar figures for both genders between 17 and 24 years of age (24.3% and 27.8%, respectively). Nevertheless, this data is consistent with that from in other studies conducted on university populations in Chile [50] (overweight in men 43.8% and women 32.4%; obesity in men 23.2% and women 21.5%), Spain [11] (25% of men with overweight or obesity compared to 13.9% of women), and the United States [12], where 41.3% of the men were obese or overweight compared to 29.9% of the women.

As far as the behavioral risk factors associated with overweight/obesity, our data indicates that male students with obesity or overweight were older, less physically active, had a worse diet (but non-significant) and admitted to more frequent drug use, particularly analgesics and tranquilizers with or without prescription, and also, although to a lesser extent, drugs like cocaine, heroin, or ecstasy.

On the other hand, in the group of female participants, it was also found that obesity and overweight were related to a older age and a more abusive use of drugs defined as medium frequency (analgesics and tranquilizers with or without a prescription).

The positive correlation found between BMI and age is a connection that appears unequivocally in most investigations conducted in this field [23,24]. In relation to physical activity, the male students had a higher mean of frequency of physical activity than the female students. Our data indicate that this variable was related to the BMI among the male participants, whereas this was not the case among the female participants. Although it seems logical to think that the obese or overweight participants are less active than those of normal weight, we found contradictory results in the scientific literature reviewed [51]. Some studies did not find this link either for male or female students despite showing that the men are the group that does more physical exercise in their free time [24], whereas others indicated that the relation between BMI and physical activity are relevant only among the men [21,52,53]. Independent of the differences found in the data in different studies, it seems that the link between obesity and a sedentary lifestyle has been established [1,12,19,21,22,52]. In any case, our data add the idea that the student’s gender is a relevant variable we must consider when designing strategies to promote physical activity and sports in order to confront and avoid the possible health risks derived from physical inactivity.

Although in the general sample there were no differences between the men and women with respect to the diet quality index, our results indicate that diet quality is associated with overweight and obesity in the population studied, particularly among the men. These data are consistent with the literature, which has described the effects of diet quality on health, as well as the association between diet quality and overweight and obesity, indicating that overweight or obese participants score significantly lower on these indicators compared to those of normal weight [11]. There is no real consensus with regard to gender-based differences, but Arroyo et al. [11] found in both the general sample and the male subjects that the mean score for the quality index was significantly lower in those with overweight or obesity than those of normal weight. Other studies have also reported higher scores among the men compared to the women in the mean value of a Diet Quality Index [54] and in the inadequacy of dietary habits [17].

With respect to drug use in general, the use of medium-frequency drugs, such as tranquilizers with or without a prescription and over-the-counter analgesics, correlated positively with BMI. Regarding male participants, that showed greater use and higher consumption of frequently used legal and illegal drugs (alcohol, tobacco and marijuana) and less-frequently used illegal drugs (cocaine, heroin, ecstasy, etc.). In contrast, drug use with or without prescription such as analgesics and tranquilizers were more frequent among female participants. These data are compatible with studies of the general and university populations [23,45,55].

Following Nolan [31], there is some evidence about the association between active drug uses with normal (or even low) body weight, although there are also some contradictions between studies with clinical and nonclinical populations, and when gender is considered. In this sense, one aspect that must be emphasized in our study is the link found between obesity and overweight and drug use. We found that the overweight and obese male participants showed greater use of pharmaceutical substances and less use of low-frequency drugs (such as cocaine, heroin, ecstasy, etc.), whereas the female participants with overweight problems used tobacco, alcohol and marijuana more, which are high-use drugs. These results are somewhat consistent with what was reported by a previous study, which indicated that obesity in men was associated with alcohol consumption, while in women it was associated with smoking [15,16,40]. A tentative explanation for this findings is that we did not control the presence of substance use disorders or any other mental disorders among the participants, so It is possible that some of the subjects would meet criteria for some of them and it would explain the relationship between pharmaceutical drug use and the increasing of consumption of food and ultimately to weight gain. In this sense Nolan [31] found an association between the recovery from drug dependence and the increase of consumption of food and weight gains. Anyway, regardless of this explanation, this could indicate two interesting aspects of the risk behaviors of Chilean university students. First of all, the data could indicate a change in the consumption pattern of legal drugs and marijuana among university women, matching the men’s rates of drug use. This phenomenon has already been confirmed in Spanish samples [56]. Secondly, it reflects the relation between different unhealthy behaviors: higher Body Mass Index, poor dietary control, less exercise, and greater substance use. In this sense, some authors have indicated repeatedly that risk behaviors tend to go together, compared to healthy behaviors; see Jessor [57].

It must be considered some limitations of the present study. First of all, it should be noted that there is no reference on the validation of the questionnaire used. However, it is used for and was provided by the government agency responsible for the prevention of alcohol and other drugs. Second, it must be considered a limitation that participants’ anthropometric measurements were not taken directly. Nevertheless, various works on the university population have used self-reporting [12,26,58]. Although there is a tendency to overestimate height and underestimate weight, it has been proven that an individual’s statement of their weight and height is a valid and trustworthy estimation [58,59]. Furthermore, the fact that the measurements in the present study were self-reported and therefore the data such as the BMI were related to perception and not completely objective shows advantages and information for promotion and prevention in health care. In fact, it is well known that the subjective variables involved may give us a better report of the participants’ perception and image. These variables related to beliefs, expectations, attitudes, health-related quality of life have shown a great relevance in relation to health behaviors [60-65] and can be more useful for understanding the causes for and perpetuation of health behaviors. Third, regarding drug use, the assessment of these behaviors by self-report method recommends caution in generalization of findings. The ideal alternative is to evaluate these variables through more invasive methods such as drug testing, however this method is not viable for studies with large samples. Thus, we opted to get this information through self-reported information mode showing a trend in a representative sample.

Secondly, the correlations found using the multiple regression analysis, despite being significant, must be considered weak as they may be a result of the large sample size used; nevertheless, a tendency has been shown that should be borne in mind in future studies.

Samples of other age levels are required, the results of which can extend and contrast the information that has been presented here. It is also suggested that longitudinal studies be undertaken that consider these same variables so as to be more certain of the influence of these risk behaviors on obesity, and that differential evolutionary trajectories be analyzed on the basis of age and gender. Furthermore, there are other factors, such as socioeconomic factors, amount of education, previously existing medical conditions, etc. that should be address in future studies.

In conclusion, the data of the present study can provide us with a better depiction of the variables present at the base of this population’s lifestyle aimed at future prevention strategies more than only having objective measurements. Specifically, our findings highlight that prevention and intervention policies should consider differences between men and women to improve the effectiveness of campaigns of prevention and health education on consumption and use of drugs, as well as for the promotion of healthy lifestyles. Not considering the gender differences may be one of the variables that could explain the failure of many public health campaigns. An intervention without bearing in mind individual variables, such as in this case the gender as well as the political and sociocultural differences, would help improve the effectiveness of interventions, as verified in other Latin American countries [66].

Finally, the data in this study support this thesis and underscore both the possibility that obesity and overweight and drug use may be more related than was initially thought as well as the need to extend the scope of preventive interventions to college students [67-69].

## Competing interests

The authors declare that they have no competing interests.

## Authors’ contributions

All authors contributed equally in the design of the study. PVV and JAP oversaw all aspects of its implementation, performed the statistical analysis and prepared the manuscript. WK and PVV acquired funding and permissions for the research, collected the data and assisted in drafting of the manuscript. PC and AVS gave statistical expertise, assisted in interpretation of data analysis and revised the manuscript for intellectual content. All authors read and approved the final manuscript.
